# A Finite Element Solution of Lateral Periodic Poisson–Boltzmann Model for Membrane Channel Proteins

**DOI:** 10.3390/ijms19030695

**Published:** 2018-02-28

**Authors:** Nan Ji, Tiantian Liu, Jingjie Xu, Longzhu Q. Shen, Benzhuo Lu

**Affiliations:** 1 LSEC, National Center for Mathematics and Interdisciplinary Sciences, Academy of Mathematics and Systems Science, Chinese Academy of Sciences, Beijing 100190, China; jinan14@lsec.cc.ac.cn; 2 School of Mathematical Sciences, University of Chinese Academy of Sciences, Beijing 100049, China; 3 CAEP Software Center for High Performance Numerical Simulation, Beijing 100088, China; liu_tiantian@iapcm.ac.cn; 4 School of Mathematical Sciences, University of Science and Technology of China, Hefei 230026, China; lffw@mail.ustc.edu.cn; 5 Department of Zoology, University of Cambridge, Cambridge CB2 3EJ, UK; lqshen@zoho.com

**Keywords:** laterally periodic Poisson-Boltzmann model, finite element method, membrane channel proteins, pore region, solvation

## Abstract

Membrane channel proteins control the diffusion of ions across biological membranes. They are closely related to the processes of various organizational mechanisms, such as: cardiac impulse, muscle contraction and hormone secretion. Introducing a membrane region into implicit solvation models extends the ability of the Poisson–Boltzmann (PB) equation to handle membrane proteins. The use of lateral periodic boundary conditions can properly simulate the discrete distribution of membrane proteins on the membrane plane and avoid boundary effects, which are caused by the finite box size in the traditional PB calculations. In this work, we: (1) develop a first finite element solver (FEPB) to solve the PB equation with a two-dimensional periodicity for membrane channel proteins, with different numerical treatments of the singular charges distributions in the channel protein; (2) add the membrane as a dielectric slab in the PB model, and use an improved mesh construction method to automatically identify the membrane channel/pore region even with a tilt angle relative to the *z*-axis; and (3) add a non-polar solvation energy term to complete the estimation of the total solvation energy of a membrane protein. A mesh resolution of about 0.25 Å (cubic grid space)/0.36 Å (tetrahedron edge length) is found to be most accurate in linear finite element calculation of the PB solvation energy. Computational studies are performed on a few exemplary molecules. The results indicate that all factors, the membrane thickness, the length of periodic box, membrane dielectric constant, pore region dielectric constant, and ionic strength, have individually considerable influence on the solvation energy of a channel protein. This demonstrates the necessity to treat all of those effects in the PB model for membrane protein simulations.

## 1. Introduction

The Poisson–Boltzmann (PB) equation is one of the most popular implicit models to describe the solvent effect through the Boltzmann distribution [1,2,3,4,5,6,7,8,9,10,11,12]. This non-linear elliptical partial differential equation solves the potential in the whole domain, which consists of both the solvent and solute. The solvent is represented by a continuum with a higher dielectric constant, while the solute is described with a lower dielectric constant and buried atomic fixed charges. The solution of the PB equation-based solvation model can provide the basis to obtain other interesting quantities, for example pKa values [13]; solvation free energies; and binding free energies [14,15]. These energy terms are meaningful to theoretical research including protein folding and design [16].

The analytic solutions of the PB equation are only available for cases where the computational domain of the biomolecule can be approximated by a simple shape, such as spheres and cylinders. However, in reality the shape of a biomolecule is complex and irregular. Under such circumstances, the PB equation needs to be solved numerically. Essentially, there are three main numerical techniques based on the discretization of the domain of interest into small regions: the finite difference method (FDM), the boundary element method (BEM), and the finite element method (FEM). In this paper, we employ FEM, which is based on a weak variational formulation. The unknown is approximated by a superposition of a set of basis functions [17,18,19,20]. At present, there is a lack of work on using FEM to solve the PBE for membrane proteins, especially for membrane channel proteins. This is the main task of our work.

To study membrane proteins, the membrane needs to be implemented in the PB equation framework to account for the sensitivity of the structure and how it functions in its surrounding environment. Consideration of the membrane increases the model complexity. Firstly, the position of the membrane makes the discrete grid/mesh construction more challenging, especially for membrane channel proteins. Secondly, the discrete distribution of membrane proteins on the membrane plane require more specific requirements with the boundary conditions. Effort has been made to include a membrane region in both Generalized Born methodologies [21,22,23,24] and the PB equation-based solvation models using FDM [25,26]. FDM has been used in periodic PB equation calculation for membrane proteins [27]. The work regarding finite difference grid construction explicitly identifying the pore region in the implicit membrane channel model has been explored in [28].

**Figure 1:**
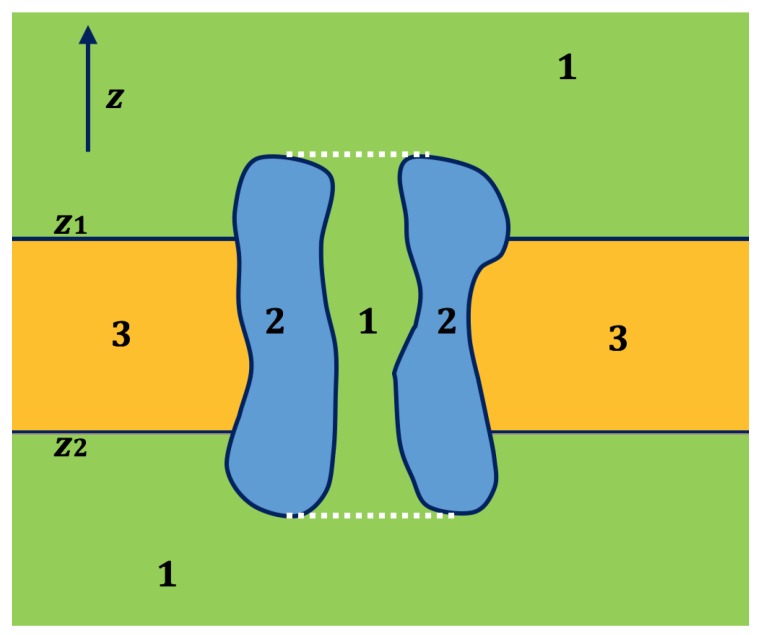
A 2D schematic picture for the cross section of an ion channel system. The solvent region is labeled 1, the membrane channel protein is labeled 2 and the membrane is labeled 3. The solvent part between the white dotted lines is the channel region [29].

The simple slab-like membrane setup is commonly used in implicit membrane solvation models. Membrane channel proteins control the diffusion of ions across biological membranes. Therefore, channels are usually end-to-end through and filled with water. Figure 1 shows a cross-section of the membrane channel system. The system consists of a membrane channel protein, a membrane region, and a solvent region. The upper and lower surfaces of the membrane are defined in the direction of *z* with the values of $z_{1}$ and $z_{2}$. The difficulty in the process of membrane construction in our FEM is to identify tetrahedrons within the channel and between $z_{2}$ and $z_{1}$. A primitive way to deal with the issue is to manually define the pore region as a combination of multiple spheres or cylinders. This method is neither efficient nor practical because any change on the radius of each membrane channel protein needs to be made by hand. In our previous work, we applied a “walk-and-detect” algorithm to directly identify the channel and, therefore, to avoid limitation above [29]. In this paper, we further expanded the algorithm to handle the membrane channel protein with a tilt angle with respect to the *z*-axis.

Introducing the membrane region into the implicit solvation model extends the capacity of the PB equation to handle membrane proteins. Usually, there are multiple proteins scattered on the membrane, therefore, a lateral periodic boundary condition can used as a good approximation to simulating the real membrane environment. The use of this type of boundary conditions can avoid boundary effects caused by the finite box size in the traditional PB calculations by using a fixed boundary potential value. In FDM, the lateral periodic boundary condition is accomplished by treating the nodes on one face of the computation grid as if they were adjacent to corresponding nodes from the opposing grid face [27]. In FEM, we mark the boundary points on one box side with the same mesh labels of the corresponding points on the opposite box side in the lateral periodic direction to achieve lateral adjacent effect (see the Method section).

The solvation energy describing the interaction between the solute and the environment can be roughly decomposed into two parts: electrostatic and non-polar solvation energies. The electrostatic contribution of the solvation free energy can be calculated with the PB equation-based implicit membrane model. The non-polar contribution of the solvation free energy is often approximated with a function that depends linearly on the solvent-accessible surface area of the molecule [30,31,32]. A similar but slightly more complex piece-wise linear model was developed [33] for non-polar energy of a protein embedded in membrane, which is incorporated into this work to calculate the total solvation energy. Non-polar solvation energy allows molecules to remain in a low-dielectric region. In contrast, the electrostatic part tends to make molecules remain in a high-dielectric region like an aqueous solvent. The final state of a molecule is a result of the balance between the non-polar solvation free energy and the electrostatic solvation free energy. In this way, we can estimate the possible tilt angle of the membrane channel protein by calculating the solvation energy in membrane environment.

## 2. Results and Discussion

### 2.1. Validation for the Treatments of Fixed Singular Charge

To evaluate the effectiveness of various treatments for fixed singular charges and compare the performance of different methods, we first solve the Poisson equation (corresponding to the zero ionic strength case in PB model) for a single atom model without membrane, which can be solved analytically. In this model, we use a sphere with a radius r=1 Å, and a unit charge $q=e_{c}$ at the center to represent the single atom solute region, which is enclosed by a bigger homocentric sphere with a radius of 200 Å as outer boundary. We adopted different dielectric constants in the solute region ($\epsilon_{m}=2$) and the solvent region ($\epsilon_{s}=80$).

The analytical form of the electrostatic solvation energy for a single atom in the bulk with an ionic concentration $c_{bi}=0$ reads

(1) $$\Delta G_{ele}=\frac{e_{c}^{2}}{8r\pi \epsilon_{0}}\left(\frac{1}{\epsilon_{m}}-\frac{1}{\epsilon_{s}}\right)$$

In this case, the electrostatic solvation energy is $\Delta G_{ele}=-80.9398$ (kcal/mol) for the parameters given above. The relative error is given by

$$E_{re}=\left|\frac{\Delta G_{ele}-\Delta G_{ele,h}}{\Delta G_{ele}}\right|\times 100\%$$

where $\Delta G_{ele,h}$ is the numerical solution of $\Delta G_{ele}$.

We explored three methods to solve the single atom model. The results are shown in Table 1. Each method occupies two rows in the table with the second row being the result of a uniformly refined mesh. For each method, we noticed an obvious improvement on the relative errors after a mesh refinement is employed. This means all three methods did well in space convergence. Among the three methods, the weighted assignment method and the direct integral method demonstrated slightly better performance than the average assignment method. In fact, the disparity may be large in membrane channel protein calculations for the accumulated errors produced by the number of atoms. We further compared the space convergence of the weighted assignment method and the direct integral method in the following.

**Figure 2:**
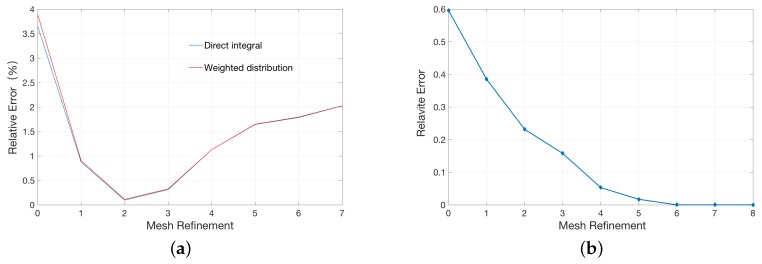
Relationship between the relative error and the times of mesh refinement: (**a**) single atom model represented by a singular point charge at the center; and (**b**) single atom model represented by a uniform charge distribution in the unit sphere.

A conventional method to improve the accuracy of numerical calculations is to continue to refine the mesh. However, this strategy may not work well to the point charge distribution as in our case due to existent singularities in the solution as showed Figure 2a. In the first several mesh refinement steps, the relative error gradually reduces as the mesh gets finer and reaches the minimum after two rounds of refinements. However, further refinement leads to a gradual increase in the relative errors, even to a greater error than one-time refinement. As a comparison, we calculate the relative error of a single atom model with a uniform charge distribution instead of a singular point charge inside the sphere in Figure 2b. As shown, mesh refinement consistently improved the accuracy of the calculations, which is in accordance with the traditional finite element analysis.

**Figure 3:**
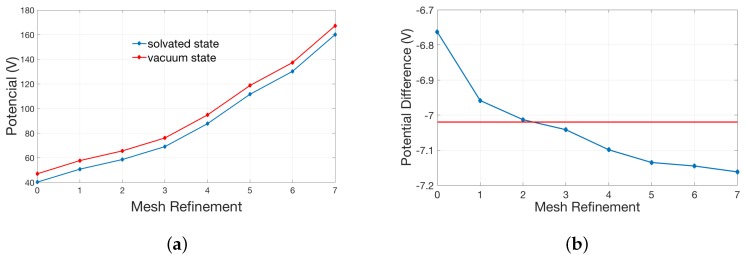
Relationship between the potential at the position of the singular charge in the single atom model and the times of mesh refinement: (**a**) potentials in the solvated state and reference state (vacuum state); and (**b**) the difference of two potentials in (**a**).

The counter-intuitive observation of finer meshes with higher errors are, in our opinion, due to singular charges. In theory, the potential at the position of the singular charge is infinite. The electrostatic solvation energy was calculated in the solvated state and reference state (vacuum state). However, this potential becomes finite in numerical simulation. We have to approximate the real solution by the refinement of the mesh. Given the relative error of two iteration potentials in the numerical calculation, the numerical magnitude of the solution is larger (which is reasonably closer to the real singularly infinite value) as the mesh becomes finer, and the absolute error of the iteration potentials become larger too. The electrostatic solvation energy may be more erroneous from calculating the difference between two larger values (the difference can be magnified by the absolute error). The final electrostatic solvation energy is the balance between the relative error and the absolute error of the iteration potentials. Please see the broken line graph Figure 3a between the potential at the position of the singular charge and times of mesh refinement.

As shown in Figure 3a, both potentials in the solvated and vacuum state gradually increase as the size of the mesh decreases. In Figure 3b, the red line represents the potential difference, corresponding to the analytic solution. It is easy to see that the potential difference gets closer to the red line in the previous refinement (especially after two times of refinements) but moves away with the further refinement. This explains the behavior in Figure 2a.

As shown in Figure 2a, the minimal relative error is obtained is obtained after two times of refinements. We thus can analyze this mesh resolution and recommend it as a reference for accurate FEM PB simulations. We calculate the average volume of the tetrahedrons elements within the concentric sphere with a radius of 5 Å, and the corresponding length of edge for an equivalent averaged regular tetrahedron is about 0.37 Å. Interestingly, this mesh resolution in FEM is found to be equivalent to a cubic grid space of 0.25 Å in FDM, which coincides well with previous observations in finite difference PB calculations.

### 2.2. Application of Lateral Periodic Boundary Condition

In Section 2.2.1, We use a DNA molecule to examine the effects of lateral periodic boundary conditions and compare different numerical methods for solution of the PB equation; In Section 2.2.2, We study the effects of the ion strength and membrane thickness on the electrostatic solvation energy of the ion channel gA, and further discuss the importance of element recognition for the channel region. In Section 2.2.3, We add the non-polar solvation energy part of the channel protein in membrane environment to estimate the proper tilt angle of the channel protein.

#### 2.2.1. Validation of Lateral Periodic Boundary Conditions

We consider a DNA molecule model possessing 778 atoms and −22 $e_{c}$ fixed charges. To apply the lateral periodic boundary conditions, we need to ensure that the box faces are symmetric in the x and y directions (the membrane plane). The parameters in this model are $\epsilon_{m}=2$ , $\epsilon_{s}=80$ and $c_{bi}=0.05$.

The presence of the singular charge distribution in Equation (4) indicates that its solution is not continuous and is actually singular. The solution obtained with the methods developed in this work as described above is in fact a continuous approximation of the real solution. Another class of methods as described in [12] is called decomposition approach. It aims to remove the singular component of the potential and to solve a left regular equation using generic numerical methods. The decomposition method can achieve accurate solution of the PB equation in the entire solute-solvent domain, but the method so far only works for non-periodic problems (charge non-neutrality inside a molecule is an issue in periodic situation, which will be addressed in the future work). Here, we will compare the decomposition method for a non-period situation with the methods used in this work. The potential decomposition method divides the potential into three parts [12]: a singular component *G*, a harmonic component *H* and a regular component $\phi_{r}$. The former two are restricted in the protein region to capture the potential property due to the singular charge distribution. $\phi_{r}$ is the solution of a regular PB equation without point charges. The corresponding electrostatic solvation energy in the decomposition method is given by

$$\Delta G_{ele}=\frac{1}{2}\sum_{i=1}^{K}q_{i}\left(H_{i}+\phi_{r,i}\right)$$

where *K* is the number of atoms in the molecule, and $\phi_{r,i}$ and $H_{i}$ are the values of $\phi_{r}$ and *H* at the *i*-th point charge, respectively.

**Figure 4:**
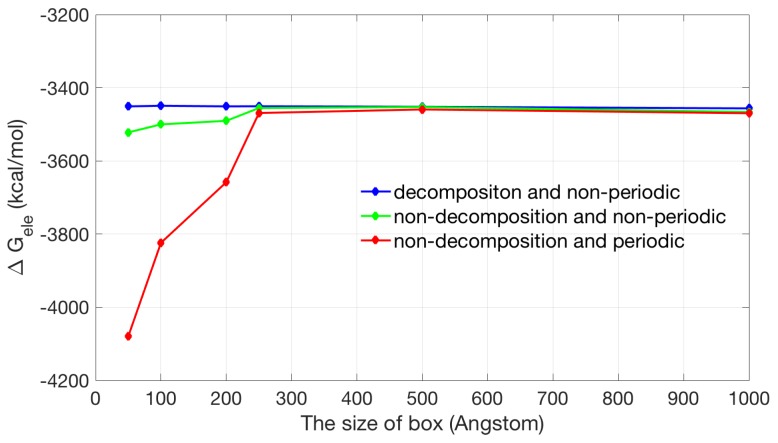
Calculation results of electrostatic solvation energies by different boundary conditions and potential calculation methods vs. the size of box. The red line denotes periodic boundary condition with non-decomposition method; the green line denotes using Dirichlet boundary condition with non-decomposition method; the blue line denotes using Dirichlet boundary condition with decomposition method.

The electrostatic energy of periodic and non-periodic systems should converge as the box size is extended toward infinity as shown in Figure 4 (red line and green line). Inspection of the plot shows that the non-periodic systems maintain a stable solvation energy in a large range of box sizes but the periodic systems do not. When the box is relatively small in the periodic case the image system has stronger effect, so electrostatic solvation energy is large (the absolute value) and as the box gets bigger and bigger, electric interaction among image system becomes weak until it is close to the result of non-periodic system. The results of the decomposition and non-decomposition methods with non-periodic boundary conditions (green line and blue line) are very close. This further verifies the feasibility and applicability of the non-decomposition method. We also compared with the result obtained with the widely used software APBS [34]. The APBS solvation energy with a box size of 50×50×50 Å${}^{3}$ and a grid space 0.25 Å is −3450.7 kcal/mol , which is in good agreement with our calculated results (−3450.9 kcal/mol with box size of 50×50×50 Å${}^{3}$ using the decomposition methods with a non-periodic boundary condition).

#### 2.2.2. Application to the Channel Protein

Gramicidin A (PDB code: 1MAG) is one of the most widely studied ion channels. It forms aqueous pores in lipid bilayers that selectively pass monovalent cations. Gramicidin A is a small 15-amino-acid β helical peptide with a narrow pore.

**Figure 5:**
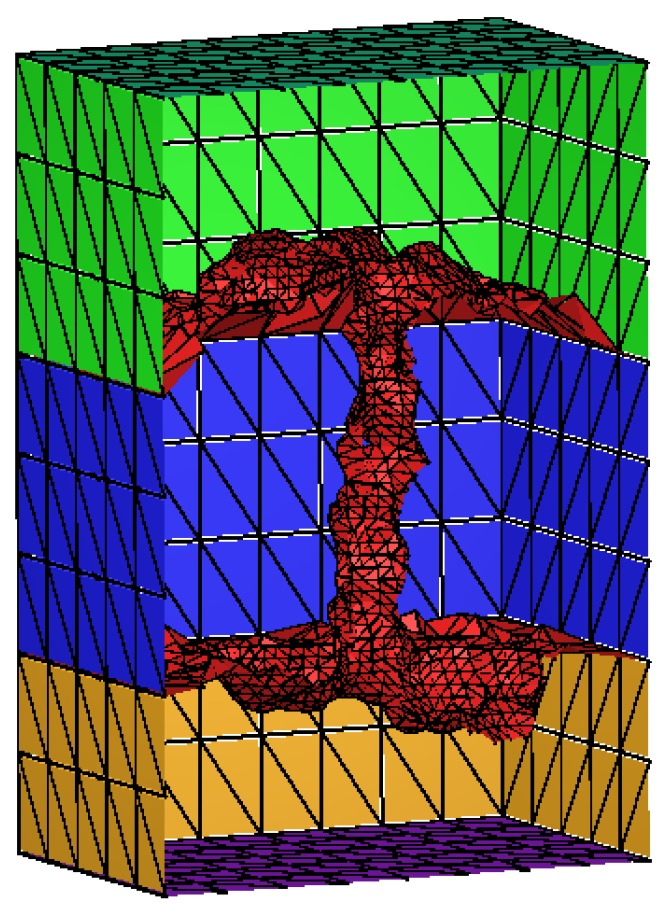
Sectional drawing of gA channel surface mesh. The molecular surface is shown in red, the membrane region is shown in blue, the solvent region is shown in green and yellow. Dark green and purple indicate the top and bottom of the box.

The triangle surface of the gA is generated by TMSmesh and the tetrahedral mesh is generated with Tetgen [35]. Figure 5 is a sectional drawing of gA channel surface mesh. The blue range represents the location of membrane (in this article, it is from −15 Å to 15 Å in *z*-axis direction). The ion channel (red) goes through the span of the membrane.

**Figure 6:**
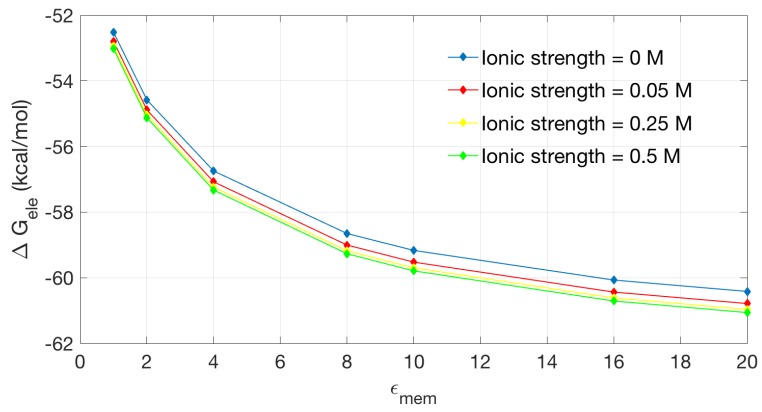
The solvation energy changes with the dielectric coefficient at the membrane region in different ionic strengths.

Figure 6 shows the relationship between electrostatic solvation energy and membrane dielectric constant. We calculate four sets of data at the ionic strength of 0, 0.05, 0.25 and 0.5 M. The four groups of data show basically a similar trend: higher $\epsilon_{mem}$ corresponds to lower solvation energy. The presence of free ions has influence on the solvation energy of the channel protein, but an increase on the ion strength does not seems to significantly affect the solvation energy.

**Figure 7:**
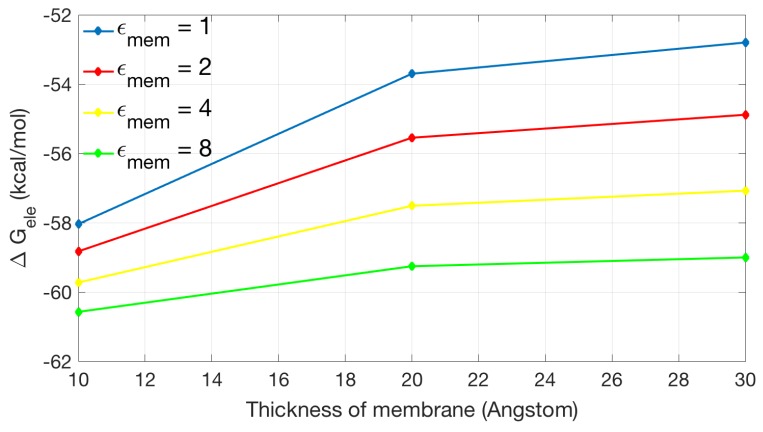
The relationship between the thickness of membrane and electrostatic solvation energy with a box size of 50×50×50 Å${}^{3}$ with period boundary condition. The relative dielectric constant in membrane is set to 1 (in blue), 2 (in red), 4 (in yellow), and 8 (in green), respectively.

Figure 7 shows the relationship between the thickness of the membrane and the electrostatic solvation energy. Different colors are used to indicate different dielectric coefficient assigned in the membrane region. In general, $\Delta G_{ele}$ increases with the increase of the membrane thickness. When $\epsilon_{mem}=1$, the difference in solvation energies from 30 to 10 Å of membrane thickness is the largest among all data, which is more than 5.3 kcal/mol. In contrast, the difference for $\epsilon_{mem}=8$ is slight (about 1.5 kcal/mol).

**Figure 8:**
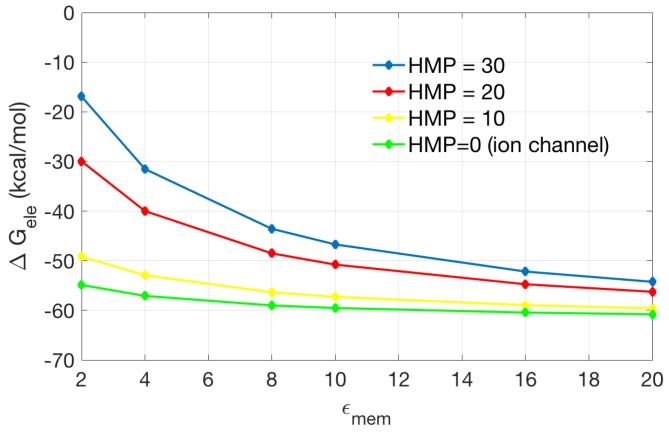
The effect of membrane in channel region on electrostatic solvation energy. The incorrectly added membrane is put into the channel region on the base of original ion channel model (in green). The thickness of the added membrane in the pore region (HMP) is 30, 20, and 10 Å, respectively. The original ion channel is equivalent to the case HMP = 0.

To explore the important role of the channel/pore recognition, we compare models containing different heights of the incorrectly added membrane in the pore region (HMP) with correctly recognized pore region (assigned with high dielectric constant as in solvent). As shown in Figure 8, when the height of the incorrectly added membrane decreases, the solvation energy is approaching that of the original channel protein (HMP = 0). When the membrane dielectric coefficients is smaller (for example, 2 or 4), the incorrectly added membrane has stronger influence on the solvation energy (inferred by the larger difference in calculated energies). This indicates that in PB modeling of membrane channel proteins, correct recognition of the pore region is important and will make considerable influence on the calculated results.

#### 2.2.3. Non-Polar Contribution to Solvation Energy

The solution of Equation (4) yields the $\Delta G_{ele}$, which accounts for the electrostatic contribution to the solvation free energy. Considering the non-polar contribution, the total solvation energy can be roughly decomposed into two parts, as shown in Equation (2):

(2) $$\Delta G_{sol}=\Delta G_{ele}+\Delta G_{np}$$

The non-polar part $\Delta G_{np}$ in the equation is usually associated with solvent accessibility (SA) and surface tension parameter γ. To consider the the non-polar term in the presence of the membrane, we adopted a modified SA-based non-polar solvation model [33]:

(3) $$\Delta G_{np}=\gamma \sum_{i=1}^{N}S\left(z_{i}\right)SA_{i}$$

where $SA_{i}$ is the solvent-accessible surface area of the *i*-th atom, γ is an empirical surface tension parameter, and S(z) introduces the variation of the surface tension along the *z* direction in the membrane environment.

**Figure 9:**
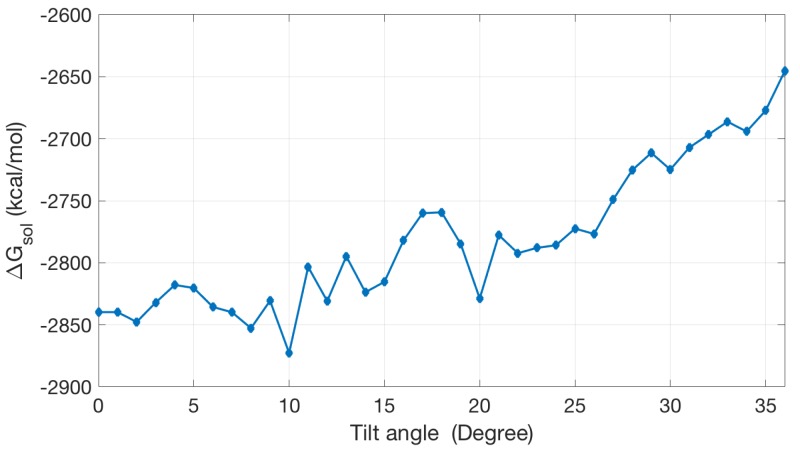
The solvation energy changes with the change of the tilt angle.

Once $\Delta G_{sol}$ is obtained, we can in principle use it to estimate the tilt angles of ion channels via searching for the lowest solvation energy with varied orientations of the channel protein. Figure 9 shows an energy curve for the dcd [36] ion channel. The calculation results show that the tilt angle can be in the range between 10 and 20 degrees, which is roughly comparable with a reported tilt angle between 20 and 30 [36]. The current PB-based solvation model for membrane protein still has a lot of room for further improvement.

## 3. Materials and Methods

### 3.1. Finite Element Method of Poisson–Boltzmann Equation

The PB equation is widely used to consider continuum electrostatic interactions in implicit solvation models. Its general form reads

(4) $$-\nabla \cdot \epsilon \nabla \phi -\lambda \sum_{i=1}^{K}c_{bi}e^{-z_{i}e_{c}\beta \phi }z_{i}e_{c}=\rho^{f}$$

where the characteristic function λ=0 in the solute region , and λ=1 in the solvent region. $\beta =\frac{1}{k_{B}T}$ is the reciprocal of Boltzmann energy composed of Boltzmann constant $k_{B}$ and the absolute temperature *T*. $c_{bi}$ is the bulk concentration of the *i*-th ion specie with valence $z_{i}$. $e_{c}$ is elementary charge. ϵ is a spatial-dependent dielectric coefficient, ϕ is the electric potential. $\rho^{f}=\sum_{j}q_{j}\delta \left(\vec{r}-\vec{r_{j}}\right)$ is the accumulation of point charges in the solute region, and $q_{j}$ is the fixed singular charge located at $\vec{r_{j}}$.

The solution of the PB equation yields the electrostatic contribution ($\Delta G_{ele}$) to the solvation free energy ($\Delta G_{sol}$). $\Delta G_{ele}$ is defined by

$$\Delta G_{ele}=G_{sys}-G_{ref}$$

where $G_{sys}$ is the electrostatic free energy of a molecule in the solvated state ($\epsilon =\epsilon_{s}$ in the solvent region and $\epsilon =\epsilon_{m}$ in the solute region) and $G_{ref}$ is the electrostatic free energy when a molecule is placed in a space with uniform dielectric constant ($\epsilon =\epsilon_{m}$) in the absence of mobile ions. In the latter condition, the second term on the left-hand side of in Equation (4) becomes zero and the Poisson–Boltzmann equation turns into a Poisson equation.

The PB equation is solved twice with corresponding parameters for $G_{sys}$ and $G_{ref}$ using a finite element method. Electrostatic solvation free energy can be calculated as

$$\Delta G_{ele}=\frac{1}{2}\sum_{i=1}^{N}q_{i}\left(\phi_{i,sys}-\phi_{i,ref}\right)$$

where $\phi_{i,sys}$ and $\phi_{i,ref}$ are the potentials at the position of the *i*-th atom in the solvated and reference states, respectively.

This paper focuses on modeling membrane channel proteins. To this end, we introduce a membrane region with a uniform dielectric coefficient into the PB model $\epsilon =\epsilon_{mem}$.

A dimensionless form of Equation (4) can be obtained by defining $u=e_{c}\beta \phi$. The weak form of Equation (4) (in the dimensionless form) in the implicit membrane solvation model is to find $u\in H_{0}^{1}\left(\Omega \right)$, so that

(5) $$\int_{\Omega }\left(\epsilon \nabla u\nabla v\right)dr^{3}-\frac{e_{c}^{2}\beta }{\epsilon_{0}}\int_{\Omega_{s}}\sum_{i=1}^{K}c_{bi}e^{-uz_{i}}vz_{i}dr^{3}=\frac{e_{c}\beta }{\epsilon_{0}}\int_{\Omega_{m}}\sum_{j}^{N}q_{j}\delta \left(\vec{r}-\vec{r_{j}}\right)vdr^{3},\;\;\;\;\forall v\in H_{0}^{1}\left(\Omega \right)$$

where Ω consists of the solute region $\Omega_{m}$, the solvent region $\Omega_{s}$ and the membrane region $\Omega_{mem}$, ϵ takes the constant $\epsilon_{m}$, $\epsilon_{s}$ and $\epsilon_{mem}$ in the corresponding region, and *u* is the dimensionless of potential ϕ by taking $u=e_{c}\beta \phi$.

Equation (5) is nonlinear. We use a Newton method with an appropriate relaxation coefficient to ensure the convergence. We take $\left\{\Phi_{j}|j=1,\ldots ,N\right\}$ as the basis functions of the finite element space and $u_{n}$ as the *n*-th Newton iterative solution of *u*. In each iteration, we need to solve the equation:

(6) $$F^{\prime }\left(u_{n}\right)\left(u_{n+1}-u_{n}\right)=-F\left(u_{n}\right)$$

where

$$\begin{array}{c} F{\left(u_{n}\right)}_{j}=\int_{\Omega }\left(\epsilon_{r}\nabla u_{n}\nabla \Phi_{j}\right)dr^{3}-\frac{e_{c}^{2}\beta }{\epsilon_{0}}\int_{\Omega_{s}}\sum_{i=1}^{K}c_{bi}e^{-u_{n}z_{i}}z_{i}\Phi_{j}dr^{3}\\ -\frac{e_{c}\beta }{\epsilon_{0}}\int_{\Omega_{m}}\sum_{j}^{N}q_{j}\delta \left(\vec{r}-\vec{r_{j}}\right)\Phi_{j}dr^{3} \end{array}$$

$$F^{\prime }{\left(u_{n}\right)}_{l,j}=\int_{\Omega }\left(\epsilon_{r}\nabla \Phi_{l}\nabla \Phi_{j}\right)dr^{3}+\frac{e_{c}^{2}\beta }{\epsilon_{0}}\int_{\Omega_{s}}\sum_{i=1}^{K}c_{bi}e^{-u_{n}z_{i}}z_{i}^{2}\Phi_{l}\Phi_{j}dr^{3}$$

Equation (4) is usually solved in a finite domain Ω subject to Dirichlet boundary conditions

(7) $$u\left(r\right)=\frac{e_{c}^{2}}{k_{B}T}\sum_{i=1}^{K}\frac{q_{i}}{\epsilon |\vec{r}-\vec{r_{j}}|}e^{-\kappa |\vec{r}-\vec{r_{j}}|/\sqrt{\epsilon }}.$$

where the square of the inverse Debye length $\kappa^{2}=\frac{2e_{c}^{2}}{k_{B}T}I_{s}$ with the the ionic strength $I_{s}=\frac{1}{2}\sum_{i=1}^{N}c_{bi}z_{i}^{2}$ and ϵ is a region-specific dielectric coefficient.

The periodic boundary condition, in fact, mimics an infinitely periodic lattice, wherein the computation grid represents the central cell [27]. In the implicit membrane solvent model, the membrane is infinitely extended along the membrane plane (x-y plane) and limited in the direction of the channel (*z* axis). Therefore, we take the Dirichlet boundary condition shown in Equation (7) on the top and bottom faces of the central box and lateral periodic boundary condition (in both directions of *x* and *y* axes) on the side faces of the central box.

**Figure 10:**
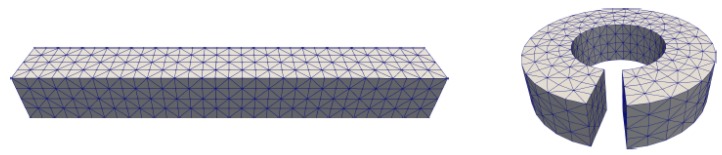
The treatment of the face gird in the direction of the periodic boundary conditions for the finite element method (FEM).

The main idea in the FEM is to represent a domain with smaller subdomains called finite elements. The distribution of the primary unknown quantity inside an element is interpolated based on the values at the nodes or at the edges. The assembly of all elements results in a global matrix system that represents the entire domain of the problem. The solution is obtained after solving the system. When dealing with Dirichlet boundary condition on the box, this constraint is applied directly to the linear system. However, for the lateral periodic boundary condition, the corresponding faces on the boundaries in the periodic direction are marked with the same label. Thus, they are treated as the same faces and shared by the two faces’ adjacent elements. In other words, the periodic boundary in FEM can be handled through “stitching” the boundary adjacent elements and treating them as normal interior elements (the boundary nodes share a same set of unknowns). The treatment mentioned above is illustrated in Figure 10.

### 3.2. Mesh Construction for Membrane Protein System

High quality surface and volume meshes form a necessary ground for numerical calculations, especially for the finite element calculation. The structures of biomolecules are highly complex. The addition of membranes increases the difficulty for the mesh construction. In this paper, we use a previously published method to generate meshes for implicit membrane solvation models containing membrane transport proteins [29]. The difficulty in the process of generating meshes for membrane protein system is to identify the elements inside a channel (see Figure 1). In our previous work, we start from an initial point inside the channel and use the “walk-and-detect” method to detect surrounding elements along the six directions of the coordinate axes. After each step, a new detection point can continue as a starting point until completing all the required elements. We can judge which element is in the channel according to the correlation of the elements in the detection path.

The limitation of the previous walk-and-detect method is the fixed detection directions. Now we improve it by walking in a random direction adaptively to adapt to the situation where the channel is not perpendicular to the membrane (x-y plane) but with a tilt angle between the *z*-axis and the channel. In addition, in order to ensure that the lateral periodic boundary conditions to be realized by FEM, the mesh of the opposite box surfaces need to be consistent (one face can be considered as a copy of the other by translation) in the periodic direction. Figure 10 shows the face grid is consistent in the direction of the periodic boundary conditions.

Our FEM uses body-fitted mesh in which the mesh needs to conform to the molecular surface. The molecular surface triangulation is a demanding task in FEM simulations. TMSmesh [37,38] and NanoShaper [39,40] are two suggested programs for general molecular surface meshing. In this paper, all the triangulated molecular surfaces are generated by either TMSmesh or NanoShaper. To ensure the consistency of boxes in the periodic direction, we prepare the box surface meshes in accordance with the boundary conditions in advance. Once a molecular surface mesh is generated, the tetrahedral volume mesh of the system-consisting of the molecule and the solvent box can be generated by the program TetGen [35].

**Figure 11:**
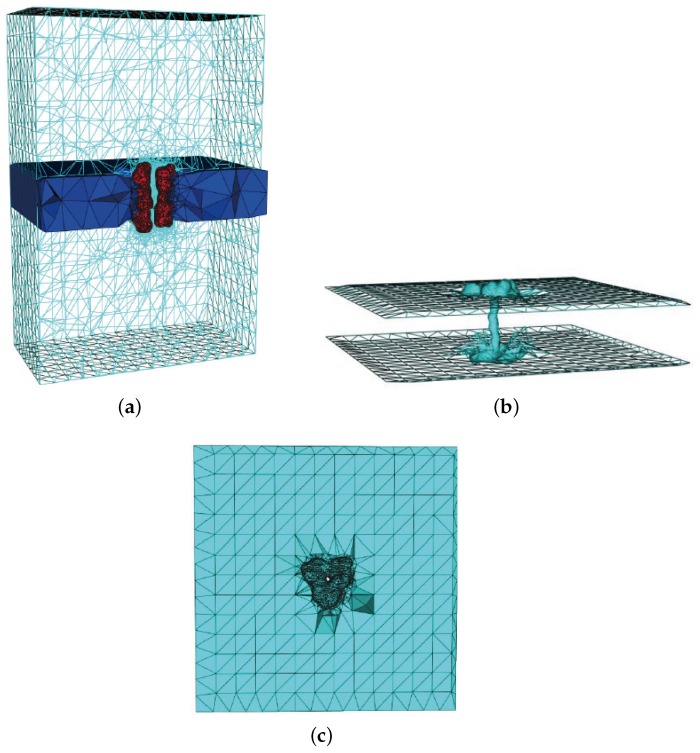
Volume mesh of gramicidin A (gA): (**a**) Wire-frame of volume mesh conforming to the boundary of a channel protein and membrane system; (**b**) the surface mesh of the membrane-protein region; and (**c**) the upper boundary surface of the membrane-protein region, in which the membrane is represented as a slab [29].

Figure 11 describes the volume mesh with emphasis on membrane-protein region of gramicidin A (gA). Gramicidin A (PDB code: 1MAG) is one of the most widely studied ion channels. It forms aqueous pores in lipid bilayers that selectively pass through monovalent cations. Gramicidin A is a small 15-amino-acid β helical peptide with a narrow pore.

### 3.3. Treatments of Fixed Singular Charges

We employ two types of methods to treat the fixed singular charges: direct integral method and charge assignment method. The latter contains three different strategies.

Direct integral method: The fixed charges are singular points which may cause numerical difficulties. As in most finite difference treatments, the singular charges are assigned to neighboring grids, which is equivalent to taking a continuous approximation of the original singular distribution. Whereas in the FEM, the singular integral in the weak form Equation (5) actually can be directly calculated as:

(8) $$\int_{\Omega_{m}}\sum_{j}^{N}q_{j}\delta \left(\vec{r}-\vec{r_{j}}\right)vdr^{3}=\sum_{j}^{N}q_{j}v\left(\vec{r_{j}}\right)$$

where the $v(\vec{r_{j}})$ is the value of *v* at the position of $\vec{r_{j}}$. It is worth noting that the numerical solution of the potential near the singular charge is sensitively determined by the mesh size due to the singularity of the real potential. We will show in the next section the mesh size-dependent accuracy in solvation energy calculations and discuss what mesh size is proper for FEM molecular simulations.

**Figure 12:**
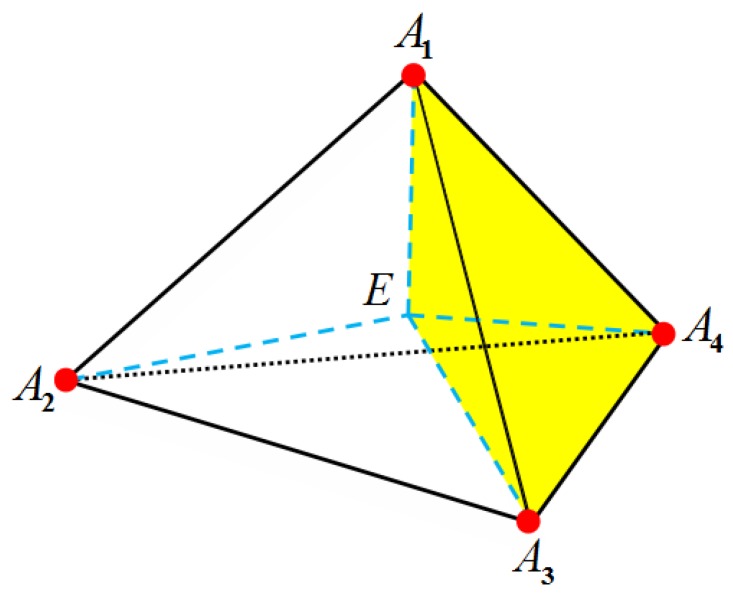
Four vertices of the tetrahedron are used as control points. *E* represents a random point inside the tetrahedron. $\lambda_{2}$ for *E* denotes the volumetric ratio of the yellow tetrahedron ($EA_{1}A_{3}A_{4}$) to the entire tetrahedron ($A_{1}A_{2}A_{3}A_{4}$). The same applies to $\lambda_{1},\lambda_{3},\lambda_{4}$.

Charge assignment to distribute the singular charges: In addition, we can also test the charge assignment methods in FEMs as in FDMs. Charge assignment is in fact to approximate the point charge distribution $\rho^{f}\left(\vec{r}\right)$ by a continuous distribution $\rho^{*}$ with $q_{j}^{*}$ denoting the assigned charge at the *j*-th node. The basic idea in the FEM is to partition a domain into a number of non-overlapping elements (we use tetrahedrons in this paper), and to approximate the solution over each element by means of a selected set of basis functions. The simplest basis functions are piecewise linear functions, in which the four vertices of a tetrahedron are used as control points. In each element, we can express the dimensionless potential *u* as

$$u\left(\vec{r}\right)=\sum_{i=1}^{4}u_{i}\lambda_{i}\left(\vec{r}\right)$$

where $u_{i}$ is the value of *u* at the *i*-th vertex of the element. The basis functions $\lambda_{i}\left(\vec{r}\right)$ have the following properties: they equal to 1 at the *i*-th vertex but 0 at the other three vertices. Once the coordinate of unknown *u* is confirmed, we can calculate the corresponding barycentric coordinates $\left\{\lambda_{1},\lambda_{2},\lambda_{3},\lambda_{4}\right\}$. In geometry, $\lambda_{i}\left(\vec{r}\right)$ denotes a volumetric ratio between the tetrahedron that encompasses $\vec{r}$ and the three vertices against the *i*-th vertex and the entire tetrahedron (see Figure 12).

Charge assignment needs to ensure integral invariance for the fixed charges. In the following, we derive three methods using piecewise linear functions as basis functions.

(1) Vertex-on-charge method: A direct thought is to constrain the mesh vertices on the fixed charges (by adding extra constrained points when using Tetgen [35]). In this way, only one basis function in an element is nonzero, so the integral value in the solute region is

$$\int_{\Omega_{m}}\rho^{*}dr^{3}=\frac{1}{4}\sum_{j=1}^{N}\left|T_{j}\right|q_{j}^{*}$$

where $|T_{j}|$ is the sum of the volumes for all the elements containing the assigned charge $q_{j}^{*}$. According to total charge invariance, the assigned charge $q_{j}^{*}=\frac{4}{|T_{j}|}q_{j}$.

(2) Average assignment method: A simple idea is to assign each charge equally to the neighboring vertices within a truncating sphere radius. Suppose a charge $p_{j}$ is enclosed by a sphere with a radius $r_{j}$. The enclose vertices are treated equally and assigned with the same charges $p^{*}$. The integral of the new distribution $\rho^{*}$ is equal to its L1-norm.

$$\int_{\Omega_{m}}\rho^{*}d\Omega =∥\rho^{*}{∥}_{1}$$

Thus, the assigned charge $q_{j}^{*}=\frac{q_{j}}{∥\rho^{*}{∥}_{1}}$.

(3) Weighted assignment method: It is unreasonable that all the nodes in the intercepting distance play the same role in the average assignment method. A weighted assignment method can be an improvement.

Inspired by the vertex-on-charge method, it is quite clear that we can assign the fixed charge $q_{i}$ to the four vertices of the element and select barycentric coordinates as weights. The larger the component of barycentric coordinates, the more charges are allocated to the corresponding vertices. Thus, the four vertices are assigned with charges $\lambda_{1}q_{i},\lambda_{2}q_{i},\lambda_{3}q_{i},\lambda_{4}q_{i}$, and the integral of this new distribution $\rho^{*}$ reads

$$\int_{\Omega_{m}}\rho^{*}d\Omega =\frac{1}{4}\sum_{i=1}^{N}(\sum_{j=1}^{4}|T_{j}\left|\lambda_{j}\right)q_{i}$$

where $|T_{j}|$ is the sum of the volumes of the tetrahedrons containing the *j*-th vertex of the *i*-th assigned charge. Therefore, all the assigned charges need to be scaled by a factor $\frac{4}{\sum_{j=1}^{4}\left|T_{j}\right|\lambda_{j}}$.

The vertex-on-charge method needs a special treatment in the process of mesh generation. Otherwise, it may cause poor mesh quality. The average assignment method does not need to determine which elements the fixed charges are in but it ignores the different influences of different related nodes. The weighted assignment method takes the difference of grid node into consideration at a computational cost. In this paper, we compared the accuracy of the latter two methods with the direct integral method in the Result section.

## 4. Conclusions

In this paper, we present a finite element method for PB electrostatics calculations of the membrane channel systems with periodical boundary condition (FEPB). To verify the correctness of the program for FEPB, especially for the treatment of the fixed singular charges, we compare three strategies in the single atom model with the analytic solution and arrive at the conclusion that proper mesh refinement can improve the computational accuracy. In the process of exploring the appropriate mesh size for the FE calculations using direct integral method to treat the fixed singular charges (without charge assignment), a mesh resolution about 0.36 Å is recommended in FEM (equivalent to a cubic grid resolution about 0.25 Å in FDM) for a nearly optimal accuracy in the mesh construction. Further refinement may increase the relative error.

The effect of lateral periodic condition is shown to be significant when the box size is at the same order of channel protein size. In the model of the DNA molecule without membrane, The results of decomposition and non-decomposition methods with non-periodic boundary condition are very close. This further verifies the feasibility and applicability of the non-decomposition method. At the same time, the results of APBS calculation are consistent with our calculations.

In the application of lateral periodic boundary conditions in the channel protein, the ion concentration in the bulk plays a relatively less important role in the solvation calculation of channel proteins relative to the thickness of membrane. Solvation energy tends to increase as the membrane gets thicker especially for low $\epsilon_{mem}$. The recognition of channel region is important because the existence of membrane in the channel significantly reduces the solvation effect.

The non-polar contribution to solvation energy in the membrane environment has also been incorporated into the PB model to calculate the total solvation energy. The estimation of the the tilt angle of the channel protein is a bit rough. More factors and more accurate non-electrostatic solvation models are needed for this type of studies.

The related meshing tools and the solver FEPB are going to be available and can be executed at the online scientific computing platform: xyzgate.com.

## Figures and Tables

**Table 1:** Calculation results from different charge assignments: average assignment method, weighted assignment method and direct integral method, for the single atom model. The two rows corresponding to each method are the results for the original mesh and a refined mesh by one uniform refinement (DOF means degree of freedom).

Singular Charges Treatments	Elements	DOFs	$G_{\mathrm{ele},h}$ (kcal/mol)	$E_{\mathrm{re}}$
Average assignment method	22614	3745	−77.01	%4.9
	92595	17037	−80.11	%1.03
Weighted assignment method	22614	3745	−77.79	%3.9
	92595	17037	−80.21	%0.90
Direct integral method	22614	3745	−77.98	%3.6
	92595	17037	−80.23	%0.87

## Abbreviations

PB	Poisson–Boltzmann
FEM	Finite element method
FDM	Finite difference method
BEM	Boundary element method
FEPB	FEM for PB electrostatics calculations of the membrane channel systems with periodical boundary conditions
TPM	The thickness of the filled membrane in the pore region
